# Cervical Cytological Findings and Vaginal Microbiota Alterations During Pregnancy: A Retrospective Analysis

**DOI:** 10.1177/30502098261471115

**Published:** 2026-07-23

**Authors:** Federica Cianfrini, Antonio d’Amati, Clelia Molinario, Belen Padial Urteta, Chiara Boccaccini, Antonio Benedetto Maria Donateo, Antonietta Vella, Rosaria Santangelo, Rosa Pasqualina De Vincenzo, Angela Santoro, Gian Franco Zannoni

**Affiliations:** 1 Anatomical Pathology Unit, Fondazione Policlinico Universitario “A. Gemelli” IRCCS, Università Cattolica S. Cuore, Rome, Italy; 2 Department of Laboratory and Infectious Diseases Sciences, Fondazione Policlinico Universitario A. Gemelli IRCCS, Rome, Italy; 3 Department of Women’s and Children’s Health and Public Health Sciences, Gynecologic Oncology Unit, Fondazione Policlinico Universitario A. Gemelli IRCCS, Rome, Italy

**Keywords:** Cytopathology, cervical cytology, pregnancy, vaginal microbiota, Pap smear

## Abstract

**Objective:**

Pregnancy induces physiological and microbiological changes in the cervicovaginal environment, which may affect the interpretation of cervical cytology. Accurate screening in this population is essential to identify both benign and pathological alterations.

**Methods:**

We conducted a retrospective monocentric study on 58 conventional Pap smears performed during pregnancy. Cytological findings were classified according to the Bethesda System, and available microbiological and HPV data were analyzed to explore potential relationships.

**Results:**

Among the 58 Pap smears, 46.6% were negative for intraepithelial lesions or malignancy (NILM), while 12.1% showed ASC-US, 10.3% LSIL, 5.2% ASC-H, and 25.9% HSIL. Candida infection and Döderlein-related cytolysis were observed in 8.6% and 8.6% of cases, respectively. HPV genotyping, available in 24 patients, identified several high-risk HPV types. Among cases with available HPV data, high-risk HPV types were more frequently observed in high-grade cytological categories, although this association did not reach statistical significance. Culture-based microbiological data were available only in a very limited subset of patients (n = 6).

**Conclusions:**

Cervical cytology during pregnancy remains a valuable screening tool, but its interpretation requires awareness of pregnancy-related changes and potential diagnostic pitfalls. The integration of HPV and microbiological data may provide additional contextual information; however, larger prospective studies with systematic testing are required to clarify the relationship between microbiota, HPV infection, and cytological abnormalities in pregnant women.

## Introduction

Pregnancy induces significant physiological and hormonal changes that profoundly affect the female genital tract, leading to notable alterations in cervical cytology and histology. Among these, the Arias-Stella reaction is a well-documented phenomenon characterized by hypertrophy and vacuolization of glandular epithelial cells, accompanied by marked nuclear pleomorphism, enlargement, and hyperchromasia. These cellular changes, while benign, can closely mimic neoplastic processes, posing diagnostic challenges in cytological evaluations during pregnancy.^1^

Concurrently, the vaginal microbiome undergoes substantial shifts throughout gestation. According to literature, any vaginal microbiome profile can be classified by five community state types (CSTs). In CST-I, II, III and V microbial communities, L. сrispatus, L. gasseri, L. iners, and L. jensenii, respectively, prevail.^2^

A predominance of Lactobacillus species, particularly Lactobacillus crispatus, is typically observed and is associated with maintaining an acidic vaginal environment that protects against vaginal dysbiosis, bacterial vaginosis (BV), aerobic vaginitis pathogenic infections, infection with human immunodeficiency virus, and different sexually transmitted diseases such as N. gonorrhoeae, G. vaginalis, and C. trachomatis. Conversely, a decreased abundance of Lactobacilli and increased microbial diversity have been linked to a higher risk of preterm birth, underscoring the critical role of the vaginal microbiome in pregnancy outcomes.^3^ In particular, an increased vaginal pH above 5.0 increases the risk of HPV infection in premenopausal women by 10 % to 20 %.^4^ Moreover, some changes in vaginal microbial community are closely related to HPV infection and CIN progression.^5^

The interpretation of Papanicolaou (Pap) smears during pregnancy is further complicated by these physiological and microbial changes. Pregnancy-related cytological changes, such as decidual cells and trophoblastic cells, can resemble atypical or dysplastic cells, leading to potential overdiagnosis or misdiagnosis. Despite these challenges, cervical cytology remains a vital tool for the early detection of cervical intraepithelial neoplasia and malignancies, even during pregnancy.^6^

Given these complexities, it is imperative to understand the spectrum of normal and abnormal cervical cytological findings in pregnant women to ensure accurate diagnosis and appropriate clinical management. While previous studies have investigated cervical cytological abnormalities and HPV infection in pregnancy, data integrating cytological findings with both HPV genotyping and concurrent microbiological assessment remain limited. In particular, the combined evaluation of cytological features and infection-related findings in a real-world clinical setting is still insufficiently characterized. In this context, the present study aims to provide a comprehensive descriptive analysis of cervical cytology during pregnancy, integrating cytological, virological, and available microbiological data, with the goal of better defining the spectrum of findings encountered in routine clinical practice.

## Materials and Methods

This retrospective, single-center study was conducted at the Anatomic Pathology Unit of Policlinico Universitario “A. Gemelli” IRCCS in Rome, Italy, aiming to evaluate cervical cytological and vaginal microbiological findings in pregnant women. This study was reviewed by the local Ethics Committee, which confirmed that, due to the retrospective nature of the study and the use of anonymized diagnostic data, formal ethical approval with an assigned code was not required. The requirement for specific informed consent was waived. All procedures were conducted in accordance with the Declaration of Helsinki (1975, revised 2013). All patients had provided general consent for the use of anonymized clinical data for research purposes.

### Study Population

Between January 2021 and December 2023, 58 pregnant women who underwent routine cervical screening at our institution were included in the study. This sample represents all consecutive pregnant women who underwent conventional cervical cytology at our institution during the study period and met the predefined inclusion criteria. No formal sample size calculation was performed due to the exploratory and retrospective nature of the study. Given the limited number of pregnant women undergoing cervical cytology within a single center, the sample size reflects real-world clinical practice rather than a predefined study design.

Inclusion criteria encompassed:

• Singleton pregnancies.
• Gestational age between 10 and 34 weeks.
• Age range of 18 to 45 years.
• No prior history of cervical intraepithelial neoplasia or cervical cancer.

Exclusion criteria were:

• Multiple gestations.
• Presence of vaginal bleeding at the time of sampling.
• Use of immunosuppressive therapy.
• Known HIV infection.

**Figure 1.**
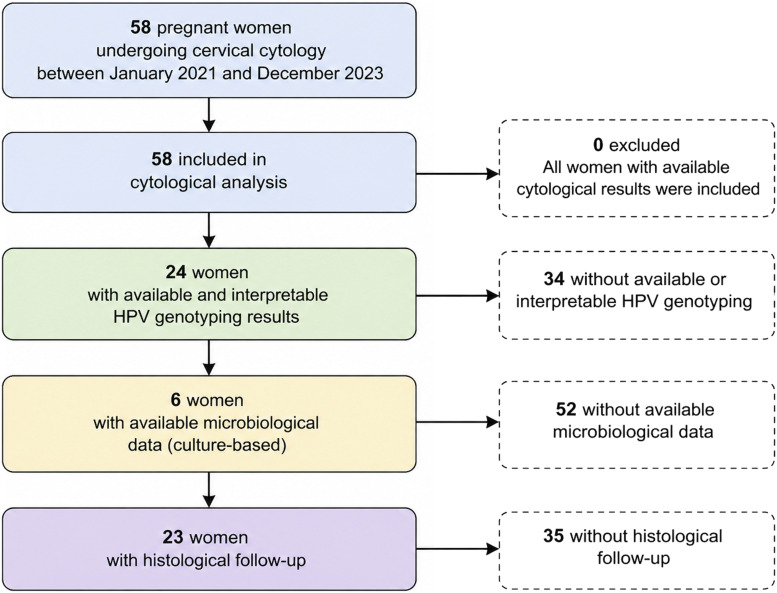
Flowchart of patient selection and data availability according to STROBE guidelines. A total of 58 pregnant women undergoing cervical cytology were included in the study. HPV genotyping results were available and interpretable in 24 cases, while culture-based microbiological data were available in 6 cases

A flowchart summarizing patient inclusion and data availability is provided in Figure 1, in accordance with STROBE recommendations.

### Cytological Sampling and Processing

Cervical samples were collected by trained obstetricians during routine prenatal visits using a cervical spatula and endocervical brush. The specimens were immediately fixed in 95% ethanol and stained using the Papanicolaou method. All cytological evaluations were performed by experienced cytopathologists and reported according to the Bethesda System 2014. Findings were categorized as:

• Negative for Intraepithelial Lesion or Malignancy (NILM)
• Atypical Squamous Cells of Undetermined Significance (ASC-US)
• Low-grade Squamous Intraepithelial Lesion (LSIL)
• High-grade Squamous Intraepithelial Lesion (HSIL)
• Atypical Squamous Cells – cannot exclude HSIL (ASC-H)

Döderlein-associated cytolysis was defined as a cytological finding characterized by the presence of abundant Lactobacillus spp. With associated epithelial cell lysis, including cytoplasmic fragmentation and numerous bare nuclei, in the absence of significant inflammatory background. This feature was assessed as part of routine cytological evaluation and not as a microbiological diagnosis.

### Microbiological Assessment

Concomitant vaginal swabs were obtained to assess the vaginal microbiota. Samples were cultured using standard microbiological techniques on selective and non-selective media, including Sabouraud Dextrose Agar (for Candida spp.), Columbia CNA Agar with 5% sheep blood (for Gram-positive bacteria), and MacConkey Agar (for Gram-negative bacteria). Gardnerella vaginalis was identified using Human Blood Bilayer Tween Agar. Trichomonas vaginalis was assessed by wet mount microscopy. Gram staining was also performed to evaluate the presence of bacterial vaginosis, defined by a shift in vaginal flora and the identification of clue cells according to Nugent criteria.

HPV genotyping was performed using a commercially available multiplex real-time PCR assay (Anyplex™ II HPV28 Detection, Seegene Inc., Seoul, South Korea), which allows simultaneous detection and genotyping of 28 HPV types, including 19 high-risk (HR) and 9 low-risk (LR) types. DNA was extracted from cervical swab specimens using the STARMag 96 X 4 Universal Cartridge kit and processed on the Microlab STARlet automated system. HPV types were assigned based on the Ct value and melting curve analysis provided by the Seegene Viewer software. HPV genotyping results were considered available only in cases with a clearly reported positive or negative result. Cases with missing or indeterminate data were excluded from the HPV-related analyses. HPV testing and microbiological assessment were not performed systematically in all patients but were available according to routine clinical practice and/or clinical indication. Therefore, HPV-related and microbiological analyses were considered exploratory and descriptive.

### Data Collection and Statistical Analysis

Demographic and clinical data, including age, gestational age at sampling, gravidity, parity, and relevant medical history, were extracted from electronic medical records. Cytological and microbiological findings were recorded and analyzed. Statistical analyses were conducted using R software (version 4.3.1, R Foundation for Statistical Computing, Vienna, Austria). Descriptive statistics were used to summarize patient characteristics and study findings. Associations between cytological abnormalities and microbiological findings were evaluated using the chi-square test or Fisher’s exact test, as appropriate. A p-value of less than 0.05 was considered statistically significant. Odds ratios (ORs) with 95% confidence intervals (CIs) were calculated to estimate the strength of association between high-risk HPV positivity and high-grade cytological abnormalities (HSIL/ASC-H).

## Results

### Cytological Findings

Fifty-eight pregnant women, with a mean age of 33.8 ± 4.9 years (range 22–44), underwent conventional cervical Pap smear screening during routine prenatal visits. Cytological examination showed that 27 (46.6%) Pap tests were classified as negative for intraepithelial lesion or malignancy (NILM).

Inflammatory and infectious cytological changes were frequently observed. Cytological findings suggestive of Candida spp. Infection were identified in 5 cases (8.6%), while cytolysis due to abundant Döderlein bacilli (indicative of a Lactobacillus-dominated vaginal microbiota) was reported in 5 cases (8.6%).

Among cytological abnormalities of squamous origin, atypical squamous cells of undetermined significance (ASC-US) were detected in 7 cases (12.1%), and low-grade squamous intraepithelial lesions (LSIL) were diagnosed in 6 cases (10.3%). Three cases (5.2%) showed atypical squamous cells where high-grade lesions could not be excluded (ASC-H). High-grade squamous intraepithelial lesions (HSIL) were reported in 15 patients (25.9%).

**Figure 2.**
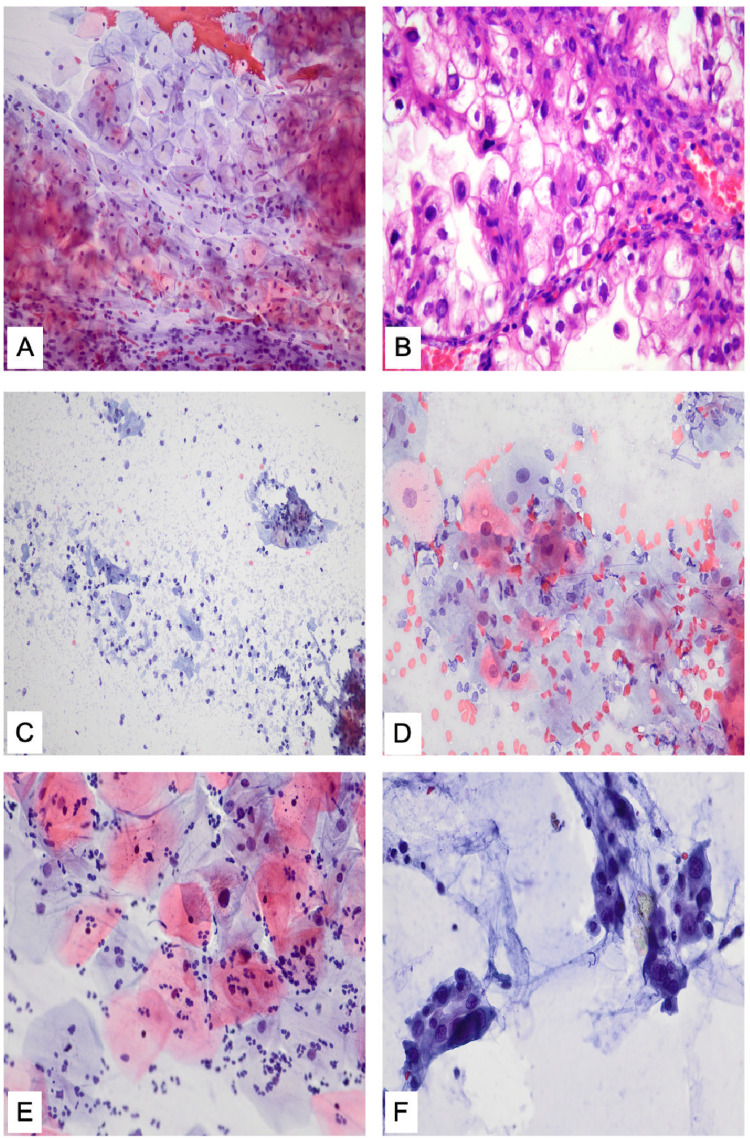
Representative morphological features observed during pregnancy. (A) Second-trimester Pap smear showing numerous navicular intermediate cells with high glycogen content (conventional Pap smear, 200×); (B) histological slide showing Arias–Stella reaction with glandular nuclear atypia and cytoplasmic vacuolization (H&E, 200×); (C) negative for intraepithelial lesion or malignancy (NILM) with cytolysis associated with Döderlein bacilli (conventional Pap smear, 100×); (D) Pap smear showing fungal elements morphologically consistent with Candida spp. (conventional Pap smear, 400×); (E) low-grade squamous intraepithelial lesion (LSIL) with koilocytosis (conventional Pap smear, 400×); (F) high-grade squamous intraepithelial lesion (HSIL) showing nuclear hyperchromasia and increased nuclear-to-cytoplasmic ratio (conventional Pap smear, 400×). NILM: Negative for Intraepithelial Lesion or Malignancy; LSIL: Low-grade Squamous Intraepithelial Lesion; HSIL: High-grade Squamous Intraepithelial Lesion

Follow-up biopsies performed during or after pregnancy confirmed significant lesions: one CIN3 case from the LSIL category, one CIN2 case from the ASC-H category, and from the HSIL group, histological assessment confirmed eight CIN3 cases, one carcinoma in situ, and one invasive cervical carcinoma. Notably, three HSIL cases initially identified during pregnancy regressed to LSIL at postpartum reassessment. The distribution of cytological diagnoses in the study cohort is summarized in Table 1. Representative cytological features for each diagnostic category are illustrated in Figure 2.

**Table 1.** Distribution of Cervical Cytological Findings in 58 Pap Smears Performed During Pregnancy. NILM: Negative for Intraepithelial Lesion or Malignancy; ASC-US: Atypical Squamous Cells of Undetermined Significance; LSIL: Low-Grade Squamous Intraepithelial Lesion; ASC-H: Atypical Squamous Cells – Cannot Exclude HSIL; HSIL: High-Grade Squamous Intraepithelial Lesion

Cytological diagnosis	Number of cases (n)	Percentage (%)
NILM	27	46.6%
ASC-US	7	12.1%
LSIL	6	10.3%
ASC-H	3	5.2%
HSIL	15	25.9%

### Microbiological Findings

Microbiological data were available only for a limited subset of patients (n = 6), and only these cases were included in microbiological analyses, while cytological evidence of Candida spp. and Döderlein-associated cytolysis was assessed in all cases as part of routine cytological evaluation. Three patients (50%) presented with a pronounced predominance of Lactobacillus species (“LATTO+”), suggestive of a physiological vaginal microbiome during pregnancy. Polymicrobial infections were identified in the other 3 patients, involving organisms such as Candida albicans, Escherichia coli, Acinetobacter baumannii, and Ureaplasma spp. Other microbiological findings included diverse bacterial populations potentially indicative of dysbiosis or infection-related inflammatory responses. Given the very limited number of cases with available microbiological data (n = 6), this analysis should be interpreted as exploratory.

### Human Papillomavirus (HPV) Genotyping Findings

**Figure 3.**
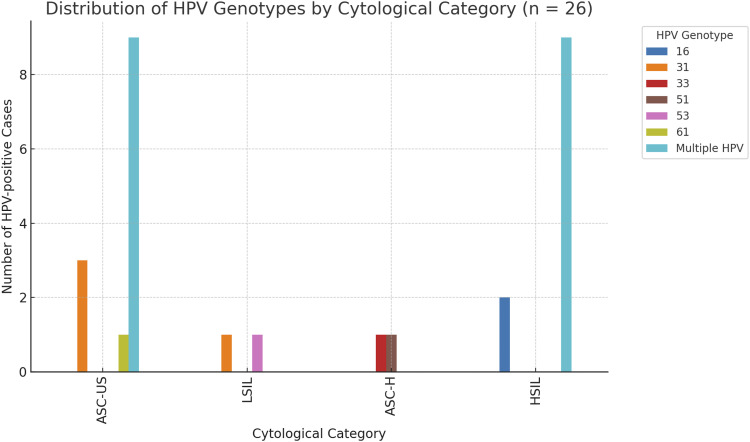
Distribution of HPV genotypes by cytological category (n = 24). Bar graph showing the frequency of selected HPV genotypes across cytological categories (ASC-US, LSIL, ASC-H, HSIL) in cases with available and interpretable HPV genotyping results. Genotypes included in the figure are those reported in the Results section (HPV 16, 31, 33, 35, 51, 53, 58, 61), while cases with multiple HPV infections are grouped under “Multiple HPV”. ASC-US: Atypical Squamous Cells of Undetermined Significance; LSIL: Low-grade Squamous Intraepithelial Lesion; ASC-H: Atypical Squamous Cells – cannot exclude HSIL; HSIL: High-grade Squamous Intraepithelial Lesion

HPV genotyping was available and interpretable in 24 out of 58 cases. Among these, 13 cases (54.2%) showed at least one high-risk HPV genotype. The most frequently detected genotype was HPV 31 (n = 4; 3 ASC-US and 1 LSIL), followed by HPV 16 (n = 7, 5 HSIL and 2 NILM). Other identified high-risk or probable high-risk genotypes included HPV 33, 35, 51, 53, and 58. Low-risk HPV types, including HPV 61, were also detected in selected cases. Multiple HPV infections were documented in 5 cases and included combinations of high-risk and low-risk genotypes. Among cases with available HPV genotyping, high-risk HPV positivity was descriptively more frequent in ASC-H/HSIL categories than in NILM/ASC-US/LSIL categories; however, this association did not reach statistical significance, likely because of the limited number of informative cases (OR = 2.45, 95% CI: 0.22–27.84; Fisher’s exact test, p = 0.615). The distribution of selected HPV genotypes by cytological category is shown in Figure 3.

### Integration of Microbiological and Cytological Findings

Because culture-based microbiological data were available for only 6 patients, these findings were analyzed descriptively and separately from cytology-based infectious or inflammatory features. Among the limited microbiologically assessed subset, Lactobacillus predominance was observed in three cases, whereas polymicrobial findings were documented in the remaining cases. These data were insufficient to establish statistically meaningful associations with cytological categories.

Cytology-based findings suggestive of Candida infection and Döderlein-associated cytolysis were recorded across the whole cohort as part of routine Pap smear evaluation. These features were observed mainly in NILM, ASC-US, and LSIL cases, whereas they were uncommon or absent in high-grade cytological categories. However, no statistically significant correlation was observed between cytological category and Candida-like changes (p = 1.00) or Döderlein-associated cytolysis (p = 0.54).

Overall, the integrated evaluation should be interpreted as exploratory and hypothesis-generating. The available data suggest that HPV genotyping may provide useful contextual information in cases with cytological abnormalities, whereas microbiological findings in this cohort are too limited to support conclusions regarding vaginal microbiota composition and cytological interpretation during pregnancy. A summary of microbiological and cytological findings is provided in Table 2. A detailed case-by-case overview is provided in the Supplemental Table.

**Table 2.** Distribution of Microbiological and HPV Findings According to Bethesda System Categories. Data Reflect the Number of Cases With Available and Interpretable Microbiological and HPV Genotyping Results. Cases With Missing or Indeterminate Data Were Excluded From the Analysis. HR-HPV: High-Risk Human Papillomavirus; LR-HPV: Low-Risk Human Papillomavirus; Co-Infections: Detection of Multiple HPV Genotypes in a Single Sample. Candida spp.: Cytological Evidence of Fungal Elements Consistent With Candida Species Observed on Pap Smear; Döderlein Cytolysis: Cytological Finding Characterized by Abundant Lactobacillus spp. Associated With Epithelial Cell Lysis

Cytological category	HR-HPV (n)	LR-HPV (n)	Co-infections (n)	Candida spp. (n)	Döderlein cytolysis (n)
NILM	1	0	0	5	5
ASC-US	3	2	1	0	0
LSIL	1	1	0	0	0
ASC-H	2	0	0	0	0
HSIL	7	2	3	0	0

## Discussion

Pregnancy is associated with hormonal and physiological changes that influence both cervical cytology and vaginal microbiota composition. Whether pregnancy increases susceptibility to HPV infection remains controversial, and reported HPV prevalence rates vary widely, ranging from 16.8% to 34.2%.^7^

In this retrospective monocentric study, we evaluated cervical cytological findings together with available HPV genotyping and microbiological data in a cohort of pregnant women, with particular attention to the diagnostic challenges posed by pregnancy-related cytological changes. Nearly half of the Pap smears (46.6%) were classified as negative for intraepithelial lesion or malignancy (NILM), supporting previous observations that benign and pregnancy-related cervical changes represent a common cytological finding in prenatal screening.^8^

Cytological evidence suggestive of Candida spp. infection and Döderlein-associated cytolysis was observed in a subset of cases. These findings are consistent with the increased prevalence of Candida spp. during pregnancy, likely related to elevated estrogen levels and glycogen-rich epithelial cells that favor yeast proliferation.^9^ Similarly, the predominance of Lactobacillus species observed in three of the six patients with available microbiological data is in line with reports describing a Lactobacillus-dominated vaginal microbiota during pregnancy and its potential role in maintaining a low vaginal pH.^10^ However, microbiological data were available for only six patients, substantially limiting the interpretability and generalizability of these observations. Therefore, the observed microbial patterns should be considered descriptive and exploratory.

The frequency of cytological abnormalities observed in our study is noteworthy. High-grade squamous intraepithelial lesions (HSIL) accounted for approximately 26% of cases, a proportion higher than that usually reported in pregnant populations, where frequencies generally range between 0.5% and 7%.^11^ This discrepancy may reflect the referral-based nature of our institution and the consequent enrichment of higher-risk patients. We also observed postpartum regression of high-grade lesions in some cases, consistent with previous studies reporting spontaneous regression of selected HSIL lesions following childbirth.^12^

HPV genotyping identified several high-risk HPV types, including HPV16, HPV31, HPV33, HPV51, HPV53, and HPV58. High-risk HPV positivity was more frequently observed among cases with high-grade cytological abnormalities; however, this association did not reach statistical significance, likely because of the limited number of cases with interpretable HPV results. Consequently, these findings should be interpreted with caution. Although descriptive, the HPV genotype distribution observed in our cohort is broadly consistent with previous studies describing the association between persistent high-risk HPV infection and cervical intraepithelial neoplasia (CIN).^13^ HPV infection has also been associated with adverse reproductive outcomes, including reduced pregnancy rates in women undergoing in vitro fertilization.^14^ Multiple HPV co-infections were observed in our cohort; however, given the limited sample size and incomplete HPV testing, no conclusions can be drawn regarding their potential clinical significance or association with lesion progression.^15^ Previous studies have suggested that HPV infection may be associated with specific vaginal microbiota patterns and increased microbial diversity.^16,17^ However, our study was not designed to characterize vaginal microbial communities, and culture-based microbiological data were available only in a very limited subset of patients. Furthermore, microbiological assessment relied on routine diagnostic methods rather than comprehensive microbiome profiling. Accordingly, our findings cannot support conclusions regarding relationships between microbiota composition, HPV infection, and cytological abnormalities during pregnancy.

The integrated evaluation of cytological findings, HPV genotyping, and available microbiological data provides a descriptive overview of concurrent alterations observed during pregnancy. In our cohort, high-grade cytological abnormalities were more frequently associated with high-risk HPV detection, whereas Candida-related changes and Döderlein-associated cytolysis were mainly observed in NILM or low-grade/inflammatory categories. However, these observations were not supported by statistically significant associations and should therefore be considered exploratory rather than confirmatory. They may represent observations warranting further investigation in larger cohorts for the interpretation of cervical cytology during pregnancy.^18^

Several limitations should be acknowledged. First, the study included a relatively small number of patients (n = 58), limiting statistical power and reducing the ability to detect potentially relevant associations. Second, microbiological data were available for only six patients and HPV genotyping was performed only in a subset of cases, substantially restricting the interpretability of subgroup analyses. Third, microbiological findings were obtained from routine clinical practice and should be considered hypothesis-generating rather than conclusive. Furthermore, the monocentric referral-based nature of our institution may have introduced selection bias, potentially contributing to the relatively high proportion of high-grade cytological abnormalities observed in this cohort. Finally, the lack of systematic HPV and microbiological testing across all patients may have introduced additional sampling bias, since these analyses were available only for selected cases.

Despite these limitations, our findings reinforce the clinical relevance of cervical cytological screening during pregnancy when interpreted within an appropriate diagnostic framework. Pregnancy-related benign changes, including the Arias–Stella reaction and decidual or trophoblastic cells, may mimic dysplastic processes and therefore require careful interpretation by experienced cytopathologists.^19^ Likewise, the evaluation of glandular abnormalities, including atypical glandular cells (AGC), remains challenging and relies heavily on clinicopathological correlation, as highlighted in previous studies.^20^

In addition to conventional cytopathological evaluation, emerging technologies such as artificial intelligence (AI) have been investigated as potential tools for improving diagnostic reproducibility. Previous studies, including our own work in placental pathology^21^ and cervical cancer screening,^22^ suggest that AI-based approaches may support cytological assessment; however, their application in pregnancy-related cervical cytology remains to be specifically validated and was not addressed in the present study.

Cervical cytology during pregnancy also retains a role in the identification of glandular abnormalities, although none were observed in our cohort. While uncommon, their recognition may have important clinical implications, particularly in pregnant women in whom diagnostic accuracy must be balanced with maternal and fetal safety.^23^ Molecular characterization of gynecological malignancies, including MMR protein status and PD-L1 expression, has demonstrated clinical relevance in other settings^24,25^; although these aspects were beyond the scope of the present study. Overall, our findings support the role of Pap testing, when interpreted within an appropriate clinical context, as a valuable tool for the detection of cervical abnormalities during pregnancy. The integration of cytological, microbiological, and HPV data may provide additional contextual information. However, the present study does not allow conclusions regarding the clinical utility of this approach. Larger prospective studies with systematic HPV testing and comprehensive microbiological assessment are required to clarify the relationships between vaginal microbiota, HPV infection, and cytological findings during pregnancy.

## Conclusions

In this retrospective single-center study, cervical cytology during pregnancy showed a broad spectrum of findings, ranging from NILM and pregnancy-related benign changes to high-grade squamous intraepithelial lesions. The relatively high proportion of HSIL observed in our cohort likely reflects the referral-based nature of our institution and should not be considered representative of the general pregnant population.

HPV genotyping, available in a subset of cases, showed high-risk HPV types in several patients with cytological abnormalities, although no statistically significant association with high-grade cytology was demonstrated. Culture-based microbiological data were available only in six cases; therefore, no firm conclusions can be drawn regarding the relationship between vaginal microbiota, HPV infection, and cytological findings.

Overall, our findings support the feasibility and clinical relevance of cervical cytology during pregnancy when interpreted by experienced cytopathologists aware of pregnancy-related diagnostic pitfalls. The integrated evaluation of cytology, HPV testing, and microbiological data remains a promising but exploratory approach that requires validation in larger prospective studies with systematic sampling and complete molecular and microbiological assessment.

## Supplemental Material

Supplemental Material - Cervical Cytological Findings and Vaginal Microbiota Alterations During Pregnancy: A Retrospective Analysis Supplemental Material for Cervical Cytological Findings and Vaginal Microbiota Alterations During Pregnancy: A Retrospective Analysis by Federica Cianfrini, Antonio d’Amati, Clelia Molinario, Belen Padial Urteta, Chiara Boccaccini, Antonio Benedetto Maria Donateo, Antonietta Vella, Rosaria Santangelo, Rosa Pasqualina De Vincenzo, Angela Santoro, Gian Franco Zannoni in Sage Open Pathology.

## Data Availability

All data generated or analyzed during this study are included in this article [and its supplementary material files]. Further enquiries can be directed to the corresponding author.
