# Supplementary material for: Cervical Cytological Findings and Vaginal Microbiota Alterations During Pregnancy: A Retrospective Analysis
Source: Sage Open Pathol. 2026 Jul 23;19:30502098261471115. doi: 10.1177/30502098261471115 (PMC13396648; doi:10.1177/30502098261471115)
Supplement: Supplemental Material - Cervical Cytological Findings and Vaginal Microbiota Alterations During Pregnancy: A Retrospective Analysis [file sj-pdf-1-pat-10.1177_30502098261471115.pdf]

Case ID	Cytology	HPV Type(s)	Microbiology
1	ASC-US	31	Mixed flora
2	ASC-US	61	Mixed flora
3	NILM	-	Mixed flora
4	LSIL	53	Mixed flora
5	ASC-US	35, 68, 61, 6, 44	Mixed flora
6	NILM	-	Mixed flora
7	NILM	-	Mixed flora
8	HSIL	-	Mixed flora
9	NILM	-	Mixed flora
10	LSIL	-	Mixed flora
11	NILM	-	Mixed flora
12	NILM	-	Mixed flora
13	HSIL	16	Mixed flora
14	NILM	-	Mixed flora
15	ASC-H	51	Mixed flora
16	ASC-H	33	Mixed flora
17	NILM	-	Candida+mixed flora
18	HSIL	-	Mixed flora
19	HSIL	NEGATIVE	Mixed flora
20	NILM	-	Candida+mixed flora
21	HSIL	58, 82, 53, 54	Mixed flora
22	NILM	-	Döderlein cytolysis+mixed flora
23	NILM	-	Candida+mixed flora
24	NILM	-	Mixed flora
25	HSIL	-	Mixed flora
26	NILM	NEGATIVE	Mixed flora
27	HSIL	16	Mixed flora
28	HSIL	-	Mixed flora
29	NILM	-	Mixed flora
30	NILM	-	Döderlein cytolysis+mixed flora
31	NILM	-	Mixed flora
32	LSIL	31	Mixed flora
33	HSIL	16, 33	Mixed flora
34	NILM	-	Mixed flora
35	NILM	-	Mixed flora
36	HSIL	-	Mixed flora
37	ASC-US	31	Mixed flora
38	NILM	-	Döderlein cytolysis+mixed flora
39	HSIL	-	Mixed flora
40	HSIL	-	Mixed flora
41	NILM	-	Döderlein cytolysis+mixed flora
42	LSIL	68, 53	Mixed flora
43	ASC-H	-	Mixed flora
44	NILM	-	Mixed flora
45	NILM	16, 53, 43	Candida+mixed flora

46	NILM	-	Döderlein cytolysis+mixed flora
47	HSIL	16, 68, 61	Mixed flora
48	LSIL	-	Mixed flora
49	HSIL	31, 53	Mixed flora
50	NILM	16	Mixed flora
51	ASC-US	51, 42, 54, 40	Mixed flora
52	HSIL	16, 68, 61	Mixed flora
53	ASC-US	18	Mixed flora
54	LSIL	21	Mixed flora
55	NILM	-	Candida+mixed flora
56	NILM	-	Mixed flora
57	NILM	-	Mixed flora
58	ASC-US	31	Mixed flora

Supplementary Table. Detailed case-by-case summary of cervical cytological diagnoses during pregnancy. The table includes the cytological interpretation according to the Bethesda System, HPV genotyping results, and microbiological findings where available. The symbol '–' indicates cases in which HPV genotyping was not performed or data were not available.
